# Molecular Mechanisms of AMH Signaling

**DOI:** 10.3389/fendo.2022.927824

**Published:** 2022-06-22

**Authors:** James A. Howard, Kaitlin N. Hart, Thomas B. Thompson

**Affiliations:** ^1^ Department of Pharmacology & Systems Physiology, University of Cincinnati, Cincinnati, OH, United States; ^2^ Department of Molecular Genetics, Biochemistry, & Microbiology, University of Cincinnati, Cincinnati, OH, United States

**Keywords:** anti-müllerian hormone (AMH), anti-müllerian hormone receptor type II (AMHR2), transforming growth factor-β (TGF-β), bone morphogenetic protein (BMP), activin, persistent müllerian duct syndrome (PMDS), cell signaling, prodomain

## Abstract

Anti-Müllerian Hormone (AMH) is a secreted glycoprotein hormone with critical roles in reproductive development and regulation. Its chemical and mechanistic similarities to members of the Transforming Growth Factor β (TGF-β) family have led to its placement within this signaling family. As a member of the TGF-β family, AMH exists as a noncovalent complex of a large N-terminal prodomain and smaller C-terminal mature signaling domain. To produce a signal, the mature domain will bind to the extracellular domains of two type I and two type II receptors which results in an intracellular SMAD signal. Interestingly, as will be discussed in this review, AMH possesses several unique characteristics which set it apart from other ligands within the TGF-β family. In particular, AMH has a dedicated type II receptor, Anti-Müllerian Hormone Receptor Type II (AMHR2), making this interaction intriguing mechanistically as well as therapeutically. Further, the prodomain of AMH has remained largely uncharacterized, despite being the largest prodomain within the family. Recent advancements in the field have provided valuable insight into the molecular mechanisms of AMH signaling, however there are still many areas of AMH signaling not understood. Herein, we will discuss what is known about the biochemistry of AMH and AMHR2, focusing on recent advances in understanding the unique characteristics of AMH signaling and the molecular mechanisms of receptor engagement.

## Introduction

Anti-Müllerian Hormone (AMH), also known as Müllerian Inhibiting Substance (MIS), was first described by Alfred Jost in 1946 as a secreted testicular factor which drove the regression of the Müllerian ducts in the male fetus (1). Importantly, dysregulation of this mechanism presents clinically as Persistent Müllerian Duct Syndrome (PMDS), in which loss of function mutations in AMH or its signaling receptor, Anti-Müllerian Hormone Receptor Type II (AMHR2), lead to persistence of Müllerian duct derivatives – uterus, fallopian tubes, cervix, and upper vagina – in males (2, 3). In women, AMH is a negative regulator of folliculogenesis and dysregulation of the signaling pathway has been implicated in two leading causes of female infertility: Polycystic Ovary Syndrome (PCOS) and Primary Ovarian Insufficiency (POI) (4). Since its initial description, more recent characterization of this hormone has provided foundational insights into our current understanding of the structure and function of AMH and its signaling pathway.

**Figure 1:**
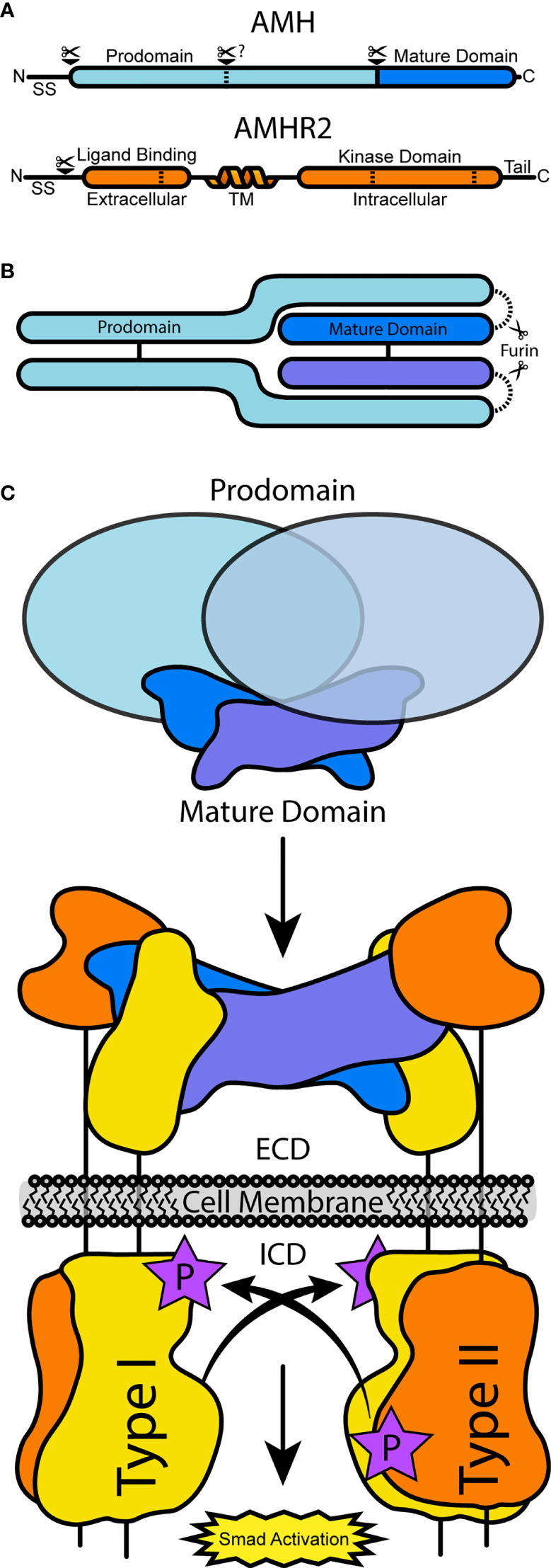
A schematic of AMH and AMHR2 processing and receptor assembly. **(A)** The full translated sequences of AMH and AMHR2 undergo processing to cleave the signal sequence. In AMH, PCs will cleave at the solid bar, separating the prodomain and mature domain, while alternative processing may occur at the dashed bar. In AMHR2, dashed bars represent alternative splicing sites. **(B)** Assembled AMH pro-complex, which may or may not be cleaved. **(C)** AMH-driven receptor assembly at the cell surface, resulting from AMH binding AMHR2 and prodomain dissociation. Type I receptors are activated and in turn activate BMP R-Smads.

AMH is a glycoprotein hormone (5) which shares structural and mechanistic homology with signaling proteins of the Transforming Growth Factor β (TGF-β) family (6). This family consists of over 30 secreted signaling ligands that have essential functions for many processes regulating cell homeostasis and human development, including reproductive development (7). These ligands are synthesized from a precursor consisting of a large N-terminal prodomain and smaller C-terminal mature signaling domain (**Figure 1A**). Folding, dimerization, and secretion are regulated by prodomains, which are cleaved from the smaller signaling domain and, in most cases, remain noncovalently associated (**Figure 1B**) (8). Ligands signal by binding to the extracellular domain (ECD) of two type I and two type II serine/threonine receptor kinases. This complex brings the intracellular kinase domain (ICD) of the constitutively active type II receptor in close enough proximity to phosphorylate the GS domain of the of the type I receptor ICD, relieving inhibition and activating Smad transcription factors (9) (**Figure 1C**). Signaling within the TGF-β family is limited to specific combinations of the seven type I receptors, Activin-like kinases 1-7 (ALK1-7), and five type II receptors, ActRIIA, ActRIIB, BMPR2, TβR2, and AMHR2 (7). It has been shown that AMH will mainly signal using ALK2 (10, 11) or ALK3 (12–14), type I receptors used by the bone morphogenetic protein (BMP) branch of TGF-B ligands, and activation of BMP R-Smads 1, 5, and 9 as well as activation of BMP reporter genes (15, 16). The other BMP type I receptor, ALK6, has a stimulatory or inhibitory effect depending on the tissue type (17, 18). AMHR2 is unique within the TGF-β family as it is the only receptor specific for a single ligand (19). In this review, we will summarize the current biochemical understanding of AMH as a TGF-β ligand from secretion to signal, with a focus on recent efforts to characterize the binding of AMH to AMHR2 and the looming gaps the field must overcome in order to better understand this important biological pathway.

## Processing and Regulation of AMH & AMHR2

AMH was first identified as a TGF-β ligand by sequence similarity of its C-terminal mature signaling domain with Activins and TGF-βs (20) and the proteolytic processing of this domain (6). The full open reading frame of human AMH (UniProtKB P03971-1) consists of a signal sequence (SS) (residues 1-24), prodomain (residues 25-451), and mature domain (residues 452-560) (**Figure 1A**) (6). Human AMH is processed canonically; mammalian proprotein convertases (PCs), such as furin, will cleave the proprotein downstream of an a R-X-X-R motif at amino acid position 448-451 to generate the 109 amino acid mature domain (21–24). Similar to other family members, PC cleavage separates the N-terminal prodomain from the C-terminal mature domain, which allows for assembly into a noncovalent complex (**Figure 1B**) (6, 25–27). Only the cleaved, processed, dimer form can properly bind its receptors and induce downstream signaling (28), but evidence of mixed circulating species of AMH suggests a regulatory role of this processing (29). Both the processed and unprocessed species are found in the serum and follicular fluid (25, 30) in varying ratios depending on age (27), sex (27), and disease state (31, 32). Interestingly, alternative cleavage products resulting from serine proteinase activity within the prodomain (**Figure 1A**) have been described during purification (6, 25, 33–35), however their biological relevance remains unknown.

The processing of AMHR2 (UniProtKB Q16671-1) is less characterized than its ligand. While there has been robust study of type II receptor regulation within the TGF-β family *via* mechanisms of internalization (36–40), homo- and heteromeric complex formation (41–43), and glycosylation (44, 45), for AMHR2, the understanding of regulation is currently limited to biosynthetic processing and surface presentation alone (46, 47). Unlike other type II receptors, it has been suggested that functional presentation of AMHR2 at the membrane is negatively regulated by cleavage or by disulfide-linked oligomerization of the extracellular domain, leading to increased retention within the ER (46). Additionally, those functional receptors that are presented appear to organize in clusters of homo-oligomers, resulting in a lack of lateral mobility (46). In mammals, receptor splice variants have been identified that result in the deletion of either amino acids 377-471 (Amhr2Δ9/10) within the kinase domain, or 17-77 (Amhr2Δ2) within the extracellular domain (47–49) (**Figure 1A**). Although their mRNA expression level in the testes and brain is 5% or less of the normal receptor, the existence of these variants raises interesting questions about their regulatory function in the AMH signaling pathway (47). Thus, continued investigation of the functional consequences of these or other splice variants is necessary to understand their potential impact on signaling. Lastly, Unlike other TGF-β ligands, investigations into mechanisms of extracellular antagonism of AMH have not been definitive (50–52). Nevertheless, regulation of ligands by protein antagonists represents a significant feature of TGF-β ligands, and the lack of known AMH-binding proteins is either a missing piece of the known mechanism or an interesting aberration from other family ligands.

## The Role of the AMH Prodomain

It is widely accepted that the prodomains of TGF-β ligands are required for proper folding and dimerization of the mature signaling ligand (53–55). While most prodomains are similar in size, an indication of secondary structure elements, there are exceptions. For example, GDF15 maintains the smallest prodomain of 18.5 kDa whereas AMH has evolved the largest of the prodomains at 45 kDa. Furthermore, unlike the ligands which typically have a conserved patter of cystines, the prodomains exhibit significant differences in the number and placement of cysteines, indicating structural divergence (7). For TGF-β1-3 the prodomains from two different chains are joined by a disulfide bond (56). The intermolecular disulfide bond increases the affinity of the prodomains for the mature ligand thorough avidity effects. Similarly, AMH also exhibits an interchain disulfide bond which likely increases its affinity for the mature ligand (6).

For some ligands, such as the TGF-βs, GDF8, and GDF11 the prodomain maintains the ligand in a latent state, and activation occurs thorough proteolysis (57–59) of the prodomain or an integrin-mediated stretching mechanism (56), both of which liberate the bound ligand from the prodomain. For AMH, BMPs, and other activin ligands, the prodomain does not render the ligand latent and the ligand is either thought to signal in the presence of the prodomain or that the prodomain is readily displaced by binding the signaling receptors. For AMH, the prodomain has been shown to allosterically regulate AMH binding to AMHR2 without inhibiting signal (28, 60). This mechanism is similar to the non-latent BMP7 pro-complex, however unlike AMH, the BMP7 prodomain has a weakly competitive interaction with the BMP type II receptors and unchanged type I receptor interactions (61). Further parallels might be drawn from the crystal structure of the BMP9 pro-complex bound by ALK1, which shows that the type I receptor can associate without displacing the prodomain (62), but this remains untested for AMH. Unlike most BMPs, the AMH prodomain has a 10-100 fold higher affinity for the mature ligand (K_d_ = 0.4 pM) (60). Despite this high-affinity interaction, bivalent binding to AMHR2 presented on a surface is able to disrupt interactions and attenuate binding 1000-fold (60). Displacement appears to be dependent on the avidity as neither monovalent binding nor soluble receptor are able to induce prodomain displacement. Thus, while certain ligands have high affinity for their prodomain and confer latency, many BMP ligands have lower affinity for their prodomains and are more readily displaced by receptor binding (8, 58). AMH appears somewhat unique in that it maintains a very high affinity for the ligand, but the prodomain can also be displaced by cell surface receptors. The high affinity of the prodomain of AMH suggests that the prodomain is likely to play an important role in either protecting AMH or facilitating signaling.

As mentioned, the prodomain seems to be an additional and principal factor of regulation within the signaling pathway. The prodomain is required for proper folding, homo- or heterodimerization, and secretion (7, 63–65), and the presence of PMDS mutations within the prodomain support this mechanism for AMH (2). In the serum, there is no unbound mature AMH ligand (25), suggesting a role for the prodomain in shuttling the mature domain to nearby and distant targets. The endocrine character of AMH is a robust research area, as we have yet to fully comprehend the breadth of extragonadal signaling targets (4, 18, 66, 67). For other ligands, the pro-complex also functions as a shield from extracellular antagonists (23, 33, 64, 68, 69). The interface between BMP antagonist Crossveinless 2 and BMP2 is analogous to the interface between mature BMP9 and the BMP9 prodomain (7), so the large AMH prodomain might function to protect AMH from interactions with a milieu of extracellular matrix (ECM) components. On the other hand, prodomains seem to be important for targeting the mature ligand to the cell surface through interactions with heparin (8), fibrillin (70), and other components of the ECM (7). However, unlike many BMPs, AMH does not have large positively-charged patches of amino acids which would limit its interactions with heparin; it instead has a significant hydrophobic character (71). We do know, however, that the prodomain is necessary for activity in tissue-based assays (72) but dispensable for cell-based assays (47, 52, 73). This suggests that the prodomain likely does not play a major role in the signaling mechanism but might play a larger role in the availability of the ligand by mediating ECM interactions or conferring protection from degradation or antagonism.

The prodomain itself may also be subject to regulatory mechanisms common to the TGF-β family. In this vein, the previously mentioned alternate cleavage sites of AMH (6, 74) (**Figure 1A**) might have some bearing on the activity of the noncovalent complex. Conformational changes (7), alternative cleavage (57, 75, 76), or other uncharacterized modifications (77) have been shown to prime the noncovalent complex for receptor interactions. Furthermore, there are 2 N-linked glycosylation and several possible O-linked glycosylation sites predicted within the AMH prodomain (78) comprising 13.5% of the complex mass (71). Differential modification of glycosylation may impact protein-protein interactions or cleavage, as observed in drosophila with the ortholog of BMP7, Gbb (76). Largely, we lack understanding of the regulatory role of the AMH prodomain beyond its absolute necessity for secretion and activity in the body. Whether the AMH prodomain, which is the family’s largest and most divergent, has additional function beyond increasing the availability of the AMH signaling ligand is not known. While recent advances in modeling using AlphaFold can help to visualize structure and domain architecture of the AMH prodomain (79), the lack of structural definition of this region and low homology cause difficulty in effectively modeling the AMH prodomain (62, 63, 80–82). As such, structural and biochemical characterization of the prodomain structure and its interfacing interactions with the mature ligand and receptors will help ascertain its function.

## Structural Definition of AMH and AMHR2

**Figure 2:**
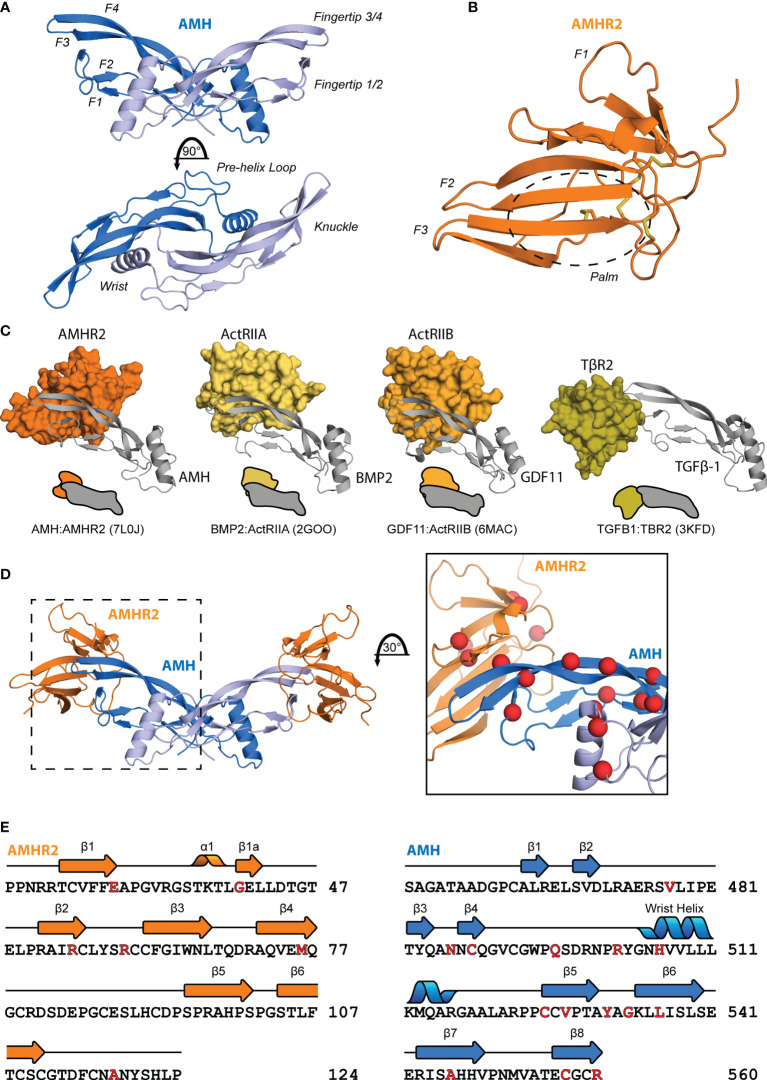
Structural features of AMH and AMHR2. Diagram of the structure of mature AMH **(A)** and AMHR2 ECD **(B)**. **(C)** Comparison of the binding modes of each ligand class to a type II receptor. **(D)** Mapping of PMDS mutations (83), indicated by red spheres, to the binary complex. **(E)** Sequence of AMH and AMHR2 annotated with secondary structure features and highlighted in red with the above PMDS mutations.

The TGF-β family is part of the cystine knot growth factor (CKGF) superfamily (7) which have a conserved fold and sequence. The overall shape can be described as an opposite-facing left and right hands in a Vulcan salute joined at the palm (**Figure 2A**). This creates a concave pocket between the wrist helix of one chain and the fingertips of the opposing chain, to which the type I receptor is recruited, and a convex surface on a single chain at the “knuckle” region, to which the type II receptor binds for Activins and BMPs (84). The extracellular ligand-binding domain of the type II receptors adopt a three-finger toxin fold, which has also classically been described with a hand-like morphology, consisting of three anti-parallel beta strand fingers and a central palm region (**Figure 2B**). These features are also conserved for AMH and AMHR2 as shown in the recently solved structure of the extracellular complex (85).

In general, ligands have evolved two central binding modes to interact specifically with their type II receptors (**Figure 2C**). Ligands of the Activin and BMP class bind at the convex, knuckle surface of the ligand fingers, while the TGF-β class ligands bind the fingertips (84). For AMH, the binding mode was unknown until the recently-solved crystal structure of AMH bound to AMHR2 (PDB:7L0J) (85). This structure provided a critical piece for understanding ligand-receptor interactions and disease-causing mutations (**Figures 2D–E**), revealing that while similar to the general binding mode of BMP and Activins, AMH utilizes a modified mode of type II receptor binding (85).

The receptor binding interaction of AMH and AMHR2 is unique within the family. While TGF-β class ligands bind TβR2 using finger 1 of the receptor and the fingertips of the ligand, Activin and BMP class ligands bind ActRIIA and ActRIIB at the palm of the receptor and the knuckles of the ligand (86–91) (**Figure 2C**). Like Activins and BMPs, AMH binds AMHR2 using the palm of the receptor and the knuckles of the ligand, however this interface is shifted towards the fingertips by about 7.5 Å. Additionally, fingers 1 and 3 of AMHR2 wrap around the ligand making unique contacts with sections of AMH not observed with Activins and BMPs, especially within fingers 3 and 4 of AMH and the connecting loop (85). While the mature ligand is similar to each of the other three classes (the root mean square deviation of the Cα positions is below 2 Å for BMP2, GDF11, and TGF-β1) the AMH ligand adopts a flat character of the fingers akin to TGF-βs.

The structure of AMH bound to AMHR2 highlighted structural differences in each that are likely responsible for specificity. Of note, AMH has a truncated finger 1/2 loop relative to other ligands that facilitates the wrap-around mechanism of AMHR2 (85). More significant variance is observed on the receptor side with differences between AMHR2 and other type II receptors undoubtedly contributing to specificity. Most notably is the conformation of finger 1 of AMHR2 which is extended compared to other receptors and forms a favorable interaction with AMH. While the number of disulfide bonds are similar, a shift of one cysteine (Cys60) results in unique structural character of AMHR2. The altered location of the disulfide bond brings together the finger 2/3 loop and finger 3 to create unique surface for AMH binding (85). These distinct conformational features of AMHR2 promote the selectivity of AMH binding and signaling.

Where we still lack critical information is in our understanding of the nature of interactions between AMH and its type I receptors. The affinity of AMH for its type II receptor has been shown to resemble TGF-βs or Activins, while affinity for the type I receptor, though not yet directly tested, is assumed to follow the same low affinity archetype as the above ligand classes (28, 73, 85). What is known is that AMH lacks two conserved tryptophan residues present in the type I receptor binding site of BMPs. Importantly, these residues have been shown to be necessary for proper signaling in members of the BMP class (92). In fact, the entire type I binding interface is dissimilar to that of BMPs (86, 93, 94) and contains more polar and charged residues, yet AMH will signal using the same type I receptors – ALK2, ALK3, ALK6 – as BMPs. It will be interesting to determine how AMH accommodates for its binding and specificity of type I receptors with these differences. It is possible that type I receptor binding is shifted relative to BMPs and might even potentially interact with the type II receptor in a cooperative mechanism similar to TGF-β, however, this has yet to be explored.

## Discussion

Recent studies have revealed a wealth of information about the molecular mechanisms of AMH signaling, but the field has a long way to go towards a full understanding of the intricacies of this unique pathway. The biochemistry of AMH is certainly less characterized than its TGF-β family counterparts. Knowledge of these structures and their interactions can help explain the expanding genetic information linked to human diseases, such as PMDS and PCOS. For example, several mutations have been identified in the prodomain, however, we lack the structural information needed to better understand how these mutations impact AMH function.

The interaction between mature AMH and the extracellular domain of AMHR2 is perhaps the most actionable piece of recent data characterizing AMH. The crystal structure demonstrates unique features that set AMH apart from other ligand classes, as well as an atomic-level explanation for PMDS mutants which map to the interface (**Figures 2D–E**). This story is, of course, incomplete without a structure of mature AMH bound by a type I receptor. While the mutual exclusivity of the AMH and AMHR2 interaction is an interesting feature within the TGF-β family, equally interesting is how the intracellular kinase domain of AMHR2 can employ type I receptors shared with BMPs yet propagate an AMH-specific signal. Intracellular interactions remain something of a black box for the fields of both TGF-β and AMH biology.

Looking ahead, further structural studies of the AMH ligand and receptor are warranted; these studies must be supported by stronger assay development. Most importantly, the field should address discrepancies between *in vitro* and *in vivo* studies, especially concerning the prodomain. It has been known for some time that the AMH prodomain is required for biological function. This feature is reflected in tissue-based assays but not in cell-based assays where the mature ligand will suffice. Better care should be taken to include the prodomain, when possible, to better replicate the biological context of AMH and allay concerns about differences between these two assay systems. Additionally, the ability to distinguish between the transcriptional outcome of AMH and BMP signaling would be a great and powerful tool for probing the mechanisms of the signaling pathway at every level. Research into this area might also help to answer a major question of AMH signal in general: is there a signaling cascade unique to AMH, or does AMH modulate a pre-existing BMP signal to generate unique outcomes? Although the research areas in need of attention are difficult, fresh data and new techniques have done wonders to answer critical questions and spark novel hypotheses about how this pathway truly functions.

## Author Contributions

JH and KH developed the concept. JH wrote the manuscript. KH and TT edited and revised the manuscript for important intellectual content. All authors read and approved the submitted version.

## Funding

This work was funded by an R01 from the Eunice Kennedy Shriver National Institute of Child Health and Human Development, R01HD105818.

## Conflict of Interest

The authors declare that the research was conducted in the absence of any commercial or financial relationships that could be construed as a potential conflict of interest.

## Publisher’s Note

All claims expressed in this article are solely those of the authors and do not necessarily represent those of their affiliated organizations, or those of the publisher, the editors and the reviewers. Any product that may be evaluated in this article, or claim that may be made by its manufacturer, is not guaranteed or endorsed by the publisher.

## References

[1] Jost A. The Age Factor in the Castration of Male Rabbit Fetuses. Proc Soc Exp Biol Med (1947) 66:302. doi: 10.3181/00379727-66-16071 18921738

[2] Belville C Van Vlijmen H Ehrenfels C Pepinsky B Rezaie AR Picard J-Y. Mutations of the Anti-Mullerian Hormone Gene in Patients With Persistent Mullerian Duct Syndrome: Biosynthesis, Secretion, and Processing of the Abnormal Proteins and Analysis Using a Three-Dimensional Model. Mol Endocrinol (2004) 18:708–21. doi: 10.1210/me.2003-0358 14673134

[3] Josso N Belville C di Clemente N Picard J-Y. AMH and AMH Receptor Defects in Persistent Müllerian Duct Syndrome. Hum Reprod Update (2005) 11:351–6. doi: 10.1093/humupd/dmi014 15878900

[4] di Clemente N Racine C Pierre A Taieb J. Anti-Müllerian Hormone in Female Reproduction. Endocr Rev (2021) 42:753–82. doi: 10.1210/endrev/bnab012 33851994

[5] Picard JY Tran D Josso N. Biosynthesis of Labelled Anti-Müllerian Hormone by Fetal Testes: Evidence for the Glycoprotein Nature of the Hormone and for its Disulfide-Bonded Structure. Mol Cell Endocrinol (1978) 12:17–30. doi: 10.1016/0303-7207(78)90098-9 720747

[6] Pepinsky RB Sinclair LK Chow EP Mattaliano RJ Manganaro TF Donahoe PK. Proteolytic Processing of Mullerian Inhibiting Substance Produces a Transforming Growth Factor-Beta-Like Fragment. J Biol Chem (1988) 263:18961–4. doi: 10.1016/S0021-9258(18)37375-7 2974034

[7] Hinck AP Mueller TD Springer TA. Structural Biology and Evolution of the TGF-β Family. Cold Spring Harb Perspect Biol (2016) 8:a022103. doi: 10.1101/cshperspect.a022103 27638177 PMC5131774

[8] Sengle G Ono RN Sasaki T Sakai LY. Prodomains of Transforming Growth Factor Beta (TGFbeta) Superfamily Members Specify Different Functions: Extracellular Matrix Interactions and Growth Factor Bioavailability. J Biol Chem (2011) 286:5087–99. doi: 10.1074/jbc.M110.188615 PMC3037620 21135108

[9] Wrana JL Attisano L Wieser R Ventura F Massagué J. Mechanism of Activation of the TGF-Beta Receptor. Nature (1994) 370:341–7. doi: 10.1038/370341a0 8047140

[10] Visser JA Olaso R Verhoef-Post M Kramer P Themmen AP Ingraham HA. The Serine/Threonine Transmembrane Receptor ALK2 Mediates Müllerian Inhibiting Substance Signaling. Mol Endocrinol (2001) 15:936–45. doi: 10.1210/mend.15.6.0645 11376112

[11] Clarke TR Hoshiya Y Yi SE Liu X Lyons KM Donahoe PK. Müllerian Inhibiting Substance Signaling Uses a Bone Morphogenetic Protein (BMP)-Like Pathway Mediated by ALK2 and Induces SMAD6 Expression. Mol Endocrinol (2001) 15:946–59. doi: 10.1210/mend.15.6.0664 11376113

[12] Jamin SP Arango NA Mishina Y Hanks MC Behringer RR. Requirement of Bmpr1a for Müllerian Duct Regression During Male Sexual Development. Nat Genet (2002) 32:408–10. doi: 10.1038/ng1003 12368913

[13] Sèdes L Leclerc A Moindjie H Cate RL Picard J-Y di Clemente N. Anti-Müllerian Hormone Recruits BMPR-IA in Immature Granulosa Cells. PloS One (2013) 8:e81551. doi: 10.1371/journal.pone.0081551 24312319 PMC3842941

[14] Gouédard L Chen YG Thevenet L Racine C Borie S Lamarre I. Engagement of Bone Morphogenetic Protein Type IB Receptor and Smad1 Signaling by Anti-Müllerian Hormone and its Type II Receptor. J Biol Chem (2000) 275:27973–8. doi: 10.1074/jbc.M002704200 10854429

[15] di Clemente N Josso N Gouédard L Belville C. Components of the Anti-Müllerian Hormone Signaling Pathway in Gonads. Mol Cell Endocrinol (2003) 211:9–14. doi: 10.1016/j.mce.2003.09.005 14656470

[16] Orvis GD Jamin SP Kwan KM Mishina Y Kaartinen VM Huang S. Functional Redundancy of TGF-Beta Family Type I Receptors and Receptor-Smads in Mediating Anti-Müllerian Hormone-Induced Müllerian Duct Regression in the Mouse1. Biol Reprod (2008) 78:994–1001. doi: 10.1095/biolreprod.107.066605 18322278 PMC4023255

[17] Belville C Jamin SP Picard J-Y Josso N di Clemente N. Role of Type I Receptors for Anti-Müllerian Hormone in the SMAT-1 Sertoli Cell Line. Oncogene (2005) 24:4984–92. doi: 10.1038/sj.onc.1208686 15897891

[18] Silva MSB Giacobini P. New Insights Into Anti-Müllerian Hormone Role in the Hypothalamic-Pituitary-Gonadal Axis and Neuroendocrine Development. Cell Mol Life Sci (2021) 78:1–16. doi: 10.1007/s00018-020-03576-x 32564094 PMC7867527

[19] Baarends WM van Helmond MJ Post M van der Schoot PJ Hoogerbrugge JW de Winter JP. A Novel Member of the Transmembrane Serine/Threonine Kinase Receptor Family is Specifically Expressed in the Gonads and in Mesenchymal Cells Adjacent to the Müllerian Duct. Development (1994) 120:189–97. doi: 10.1242/dev.120.1.189 8119126

[20] Cate RL Mattaliano RJ Hession C Tizard R Farber NM Cheung A. Isolation of the Bovine and Human Genes for Müllerian Inhibiting Substance and Expression of the Human Gene in Animal Cells. Cell (1986) 45:685–98. doi: 10.1016/0092-8674(86)90783-x 3754790

[21] Dubois CM Laprise MH Blanchette F Gentry LE Leduc R. Processing of Transforming Growth Factor Beta 1 Precursor by Human Furin Convertase. J Biol Chem (1995) 270:10618–24. doi: 10.1074/jbc.270.18.10618 7737999

[22] Constam DB Robertson EJ. Regulation of Bone Morphogenetic Protein Activity by Pro Domains and Proprotein Convertases. J Cell Biol (1999) 144:139–49. doi: 10.1083/jcb.144.1.139 PMC2148113 9885250

[23] Constam DB. Regulation of Tgfβ and Related Signals by Precursor Processing. Semin Cell Dev Biol (2014) 32:85–97. doi: 10.1016/j.semcdb.2014.01.008 24508081

[24] Nachtigal MW Ingraham HA. Bioactivation of Müllerian Inhibiting Substance During Gonadal Development by a Kex2/Subtilisin-Like Endoprotease. Proc Natl Acad Sci U.S.A. (1996) 93:7711–6. doi: 10.1073/pnas.93.15.7711 PMC38812 8755541

[25] Pankhurst MW McLennan IS. Human Blood Contains Both the Uncleaved Precursor of Anti-Mullerian Hormone and a Complex of the NH2- and COOH-Terminal Peptides. Am J Physiol Endocrinol Metab (2013) 305:E1241–1247. doi: 10.1152/ajpendo.00395.2013 24045871

[26] McLennan IS Pankhurst MW. Anti-Müllerian Hormone is a Gonadal Cytokine With Two Circulating Forms and Cryptic Actions. J Endocrinol (2015) 226:R45–57. doi: 10.1530/JOE-15-0206 26163524

[27] Pankhurst MW Chong YH McLennan IS. Relative Levels of the Proprotein and Cleavage-Activated Form of Circulating Human Anti-Müllerian Hormone are Sexually Dimorphic and Variable During the Life Cycle. Physiol Rep (2016) 4:e12783. doi: 10.14814/phy2.12783 27147497 PMC4873634

[28] di Clemente N Jamin SP Lugovskoy A Carmillo P Ehrenfels C Picard J-Y. Processing of Anti-Mullerian Hormone Regulates Receptor Activation by a Mechanism Distinct From TGF-Beta. Mol Endocrinol (2010) 24:2193–206. doi: 10.1210/me.2010-0273 PMC5417381 20861221

[29] Pankhurst MW Leathart B-LA Batchelor NJ McLennan IS. The Anti-Müllerian Hormone Precursor (proAMH) Is Not Converted to the Receptor-Competent Form (AMHN,C) in the Circulating Blood of Mice. Endocrinology (2016) 157:1622–9. doi: 10.1210/en.2015-1834 26828745

[30] Campbell BK Clinton M Webb R. The Role of Anti-Müllerian Hormone (AMH) During Follicle Development in a Monovulatory Species (Sheep). Endocrinology (2012) 153:4533–43. doi: 10.1210/en.2012-1158 22778215

[31] Peigné M Pigny P Pankhurst MW Drumez E Loyens A Dewailly D. The Proportion of Cleaved Anti-Müllerian Hormone is Higher in Serum But Not Follicular Fluid of Obese Women Independently of Polycystic Ovary Syndrome. Reprod BioMed Online (2020) 41:1112–21. doi: 10.1016/j.rbmo.2020.07.020 33046375

[32] Pankhurst MW Shorakae S Rodgers RJ Teede HJ Moran LJ. Efficacy of Predictive Models for Polycystic Ovary Syndrome Using Serum Levels of Two Antimüllerian Hormone Isoforms (proAMH and AMHN,C). Fertil Steril (2017) 108:851–857.e2. doi: 10.1016/j.fertnstert.2017.08.012 29079276

[33] Akiyama T Marqués G Wharton KA. A Large Bioactive BMP Ligand With Distinct Signaling Properties is Produced by Alternative Proconvertase Processing. Sci Signal (2012) 5:ra28. doi: 10.1126/scisignal.2002549 22472650 PMC3699424

[34] Ragin RC Donahoe PK Kenneally MK Ahmad MF MacLaughlin DT. Human Müllerian Inhibiting Substance: Enhanced Purification Imparts Biochemical Stability and Restores Antiproliferative Effects. Protein Expr Purif (1992) 3:236–45. doi: 10.1016/1046-5928(92)90020-w 1392620

[35] Almeida J Ball BA Conley AJ Place NJ Liu IKM Scholtz EL. Biological and Clinical Significance of Anti-Müllerian Hormone Determination in Blood Serum of the Mare. Theriogenology (2011) 76:1393–403. doi: 10.1016/j.theriogenology.2011.06.008 21798581

[36] Chen Y-G. Endocytic Regulation of TGF-β Signaling. Cell Res (2009) 19:58–70. doi: 10.1038/cr.2008.315 19050695

[37] Chen C-L Hou W-H Liu I-H Hsiao G Huang SS Huang JS. Inhibitors of Clathrin-Dependent Endocytosis Enhance Tgfβ Signaling and Responses. J Cell Sci (2009) 122:1863–71. doi: 10.1242/jcs.038729 PMC2684837 19461075

[38] Di Guglielmo GM Le Roy C Goodfellow AF Wrana JL. Distinct Endocytic Pathways Regulate TGF-β Receptor Signalling and Turnover. Nat Cell Biol (2003) 5:410–21. doi: 10.1038/ncb975 12717440

[39] Hartung A Bitton-Worms K Rechtman MM Wenzel V Boergermann JH Hassel S. Different Routes of Bone Morphogenic Protein (BMP) Receptor Endocytosis Influence BMP Signaling. Mol Cell Biol (2006) 26(20):7791–805. doi: 10.1128/MCB.00022-06 PMC1636853 16923969

[40] Yao D Ehrlich M Henis YI Leof EB. Transforming Growth Factor-β Receptors Interact With AP2 by Direct Binding to β2 Subunit. MBoC (2002) 13:4001–12. doi: 10.1091/mbc.02-07-0104 PMC133610 12429842

[41] Ehrlich M Horbelt D Marom B Knaus P Henis YI. Homomeric and Heteromeric Complexes Among TGF-β and BMP Receptors and Their Roles in Signaling. Cell Signalling (2011) 23:1424–32. doi: 10.1016/j.cellsig.2011.04.004 21515362

[42] Ehrlich M Gutman O Knaus P Henis YI. Oligomeric Interactions of TGF-β and BMP Receptors. FEBS Lett (2012) 586:1885–96. doi: 10.1016/j.febslet.2012.01.040 22293501

[43] Nohe A Hassel S Ehrlich M Neubauer F Sebald W Henis YI. The Mode of Bone Morphogenetic Protein (BMP) Receptor Oligomerization Determines Different BMP-2 Signaling Pathways*. J Biol Chem (2002) 277:5330–8. doi: 10.1074/jbc.M102750200 11714695

[44] Kim Y-W Park J Lee H-J Lee S-Y Kim S-J. TGF-β Sensitivity is Determined by N-Linked Glycosylation of the Type II TGF-β Receptor. Biochem J (2012) 445:403–11. doi: 10.1042/BJ20111923 PMC3462611 22571197

[45] Partridge EA Le Roy C Di Guglielmo GM Pawling J Cheung P Granovsky M. Regulation of Cytokine Receptors by Golgi N-Glycan Processing and Endocytosis. Science (2004) 306:120–4. doi: 10.1126/science.1102109 15459394

[46] Hirschhorn T di Clemente N Amsalem AR Pepinsky RB Picard J-Y Smorodinsky NI. Constitutive Negative Regulation in the Processing of the Anti-Mullerian Hormone Receptor II. J Cell Sci (2015) 128:1352–64. doi: 10.1242/jcs.160143 25663701

[47] Imhoff FM Yang D Mathew SF Clarkson AN Kawagishi Y Tate WP. The Type 2 Anti-Müllerian Hormone Receptor has Splice Variants That are Dominant-Negative Inhibitors. FEBS Lett (2013) 587:1749–53. doi: 10.1016/j.febslet.2013.04.014 23624077

[48] Faure E Gouédard L Imbeaud S Cate R Picard J-Y Josso N. Mutant Isoforms of the Anti-Müllerian Hormone Type II Receptor Are Not Expressed at the Cell Membrane*. J Biol Chem (1996) 271:30571–5. doi: 10.1074/jbc.271.48.30571 8940028

[49] di Clemente N Wilson C Faure E Boussin L Carmillo P Tizard R. Cloning, Expression, and Alternative Splicing of the Receptor for Anti-Müllerian Hormone. Mol Endocrinol (1994) 8:1006–20. doi: 10.1210/mend.8.8.7997230 7997230

[50] Gipson GR Goebel EJ Hart KN Kappes EC Kattamuri C McCoy JC. Structural Perspective of BMP Ligands and Signaling. Bone (2020) 140:115549. doi: 10.1016/j.bone.2020.115549 32730927 PMC7502536

[51] Nilsson EE Larsen G Skinner MK. Roles of Gremlin 1 and Gremlin 2 in Regulating Ovarian Primordial to Primary Follicle Transition. Reproduction (2014) 147:865–74. doi: 10.1530/REP-14-0005 PMC4043579 24614542

[52] Kawagishi Y Pankhurst MW Nakatani Y McLennan IS. Anti-Müllerian Hormone Signaling is Influenced by Follistatin 288, But Not 14 Other Transforming Growth Factor Beta Superfamily Regulators. Mol Reprod Dev (2017) 84:626–37. doi: 10.1002/mrd.22828 28500669

[53] Gentry LE Nash BW. The Pro Domain of Pre-Pro-Transforming Growth Factor Beta 1 When Independently Expressed Is a Functional Binding Protein for the Mature Growth Factor. Biochemistry (1990) 29:6851–7. doi: 10.1021/bi00481a014 2397217

[54] Gray AM Mason AJ. Requirement for Activin A and Transforming Growth Factor–Beta 1 Pro-Regions in Homodimer Assembly. Science (1990) 247:1328–30. doi: 10.1126/science.2315700 2315700

[55] Walton KL Makanji Y Wilce MC Chan KL Robertson DM Harrison CA. A Common Biosynthetic Pathway Governs the Dimerization and Secretion of Inhibin and Related Transforming Growth Factor Beta (TGFbeta) Ligands. J Biol Chem (2009) 284:9311–20. doi: 10.1074/jbc.M808763200 PMC2666583 19193648

[56] Shi M Zhu J Wang R Chen X Mi L Walz T. Latent TGF-β Structure and Activation. Nature (2011) 474:343–9. doi: 10.1038/nature10152 PMC4717672 21677751

[57] McCoy JC Goebel EJ Thompson TB. Characterization of Tolloid-Mediated Cleavage of the GDF8 Procomplex. Biochem J (2021) 478(9):1733–47. doi: 10.1042/BCJ20210054 PMC8670536 33876824

[58] Wolfman NM McPherron AC Pappano WN Davies MV Song K Tomkinson KN. Activation of Latent Myostatin by the BMP-1/Tolloid Family of Metalloproteinases. Proc Natl Acad Sci U.S.A. (2003) 100:15842–6. doi: 10.1073/pnas.2534946100 PMC307655 14671324

[59] Walker RG Poggioli T Katsimpardi L Buchanan SM Oh J Wattrus S. Biochemistry and Biology of GDF11 and Myostatin: Similarities, Differences, and Questions for Future Investigation. Circ Res (2016) 118:1125–41. doi: 10.1161/CIRCRESAHA.116.308391 PMC4818972 27034275

[60] Cate RL di Clemente N Racine C Groome NP Pepinsky RB Whitty A. The Anti-Müllerian Hormone Prodomain Is Displaced From the Hormone/Prodomain Complex Upon Bivalent Binding to the Hormone Receptor. J Biol Chem (2022) 298:101429. doi: 10.1016/j.jbc.2021.101429 34801555 PMC8801479

[61] Sengle G Ono RN Lyons KM Bächinger HP Sakai LY. A New Model for Growth Factor Activation: Type II Receptors Compete With the Prodomain for BMP-7. J Mol Biol (2008) 381:1025–39. doi: 10.1016/j.jmb.2008.06.074 PMC2705212 18621057

[62] Salmon RM Guo J Wood JH Tong Z Beech JS Lawera A. Molecular Basis of ALK1-Mediated Signalling by BMP9/BMP10 and Their Prodomain-Bound Forms. Nat Commun (2020) 11:1621. doi: 10.1038/s41467-020-15425-3 32238803 PMC7113306

[63] Zhao B Xu S Dong X Lu C Springer TA. Prodomain-Growth Factor Swapping in the Structure of Pro-TGF-β1. J Biol Chem (2018) 293:1579–89. doi: 10.1074/jbc.M117.809657 PMC5798290 29109152

[64] Harrison CA Al-Musawi SL Walton KL. Prodomains Regulate the Synthesis, Extracellular Localisation and Activity of TGF-β Superfamily Ligands. Growth Factors (2011) 29:174–86. doi: 10.3109/08977194.2011.608666 21864080

[65] Neugebauer JM Kwon S Kim H-S Donley N Tilak A Sopory S. The Prodomain of BMP4 is Necessary and Sufficient to Generate Stable BMP4/7 Heterodimers With Enhanced Bioactivity. vivo Proc Natl Acad Sci U.S.A. (2015) 112:E2307–2316. doi: 10.1073/pnas.1501449112 PMC4426409 25902523

[66] Malone SA Papadakis GE Messina A Mimouni NEH Trova S Imbernon M. Defective AMH Signaling Disrupts GnRH Neuron Development and Function and Contributes to Hypogonadotropic Hypogonadism. Elife (2019) 8:e47198. doi: 10.7554/eLife.47198 31291191 PMC6620045

[67] Tata B El Houda Mimouni N Barbotin A-L Malone SA Loyens A Pigny P. Elevated Prenatal Anti-Müllerian Hormone Reprograms the Fetus and Induces Polycystic Ovary Syndrome in Adulthood. Nat Med (2018) 24:834–46. doi: 10.1038/s41591-018-0035-5 PMC6098696 29760445

[68] Cui Y Hackenmiller R Berg L Jean F Nakayama T Thomas G. The Activity and Signaling Range of Mature BMP-4 Is Regulated by Sequential Cleavage at Two Sites Within the Prodomain of the Precursor. Genes Dev (2001) 15:2797–802. doi: 10.1101/gad.940001 PMC312809 11691831

[69] Ashe HL Levine M. Local Inhibition and Long-Range Enhancement of Dpp Signal Transduction by Sog. Nature (1999) 398:427–31. doi: 10.1038/18892 10201373

[70] Gregory KE Ono RN Charbonneau NL Kuo C-L Keene DR Bächinger HP. The Prodomain of BMP-7 Targets the BMP-7 Complex to the Extracellular Matrix. J Biol Chem (2005) 280:27970–80. doi: 10.1074/jbc.M504270200 15929982

[71] Picard JY Goulut C Bourrillon R Josso N. Biochemical Analysis of Bovine Testicular Anti-Müllerian Hormone. FEBS Lett (1986) 195:73–6. doi: 10.1016/0014-5793(86)80133-8 3753687

[72] Wilson CA di Clemente N Ehrenfels C Pepinsky RB Josso N Vigier B. Mullerian Inhibiting Substance Requires its N-Terminal Domain for Maintenance of Biological Activity, A Novel Finding Within the Transforming Growth Factor-Beta Superfamily. Mol Endocrinol (1993) 7:247–57. doi: 10.1210/mend.7.2.8469238 8469238

[73] Hart KN Pépin D Czepnik M Donahoe PK Thompson TB. Mutational Analysis of the Putative Anti-Müllerian Hormone (AMH) Binding Interface on its Type II Receptor, Amhr2. Endocrinology (2020) 161:bqaa066. doi: 10.1210/endocr/bqaa066 32333774 PMC7286617

[74] Picard JY Josso N. Purification of Testicular Anti-Müllerian Hormone Allowing Direct Visualization of the Pure Glycoprotein and Determination of Yield and Purification Factor. Mol Cell Endocrinol (1984) 34:23–9. doi: 10.1016/0303-7207(84)90155-2 6546551

[75] Tilak A Nelsen SM Kim H-S Donley N McKnite A Lee H. Simultaneous Rather Than Ordered Cleavage of Two Sites Within the BMP4 Prodomain Leads to Loss of Ligand in Mice. Development (2014) 141:3062–71. doi: 10.1242/dev.110130 PMC4197676 24993941

[76] Anderson EN Wharton KA. Alternative Cleavage of the Bone Morphogenetic Protein (BMP), Gbb, Produces Ligands With Distinct Developmental Functions and Receptor Preferences. J Biol Chem (2017) 292:19160–78. doi: 10.1074/jbc.M117.793513 PMC5702660 28924042

[77] Pierre A Racine C Rey RA Fanchin R Taieb J Cohen-Tannoudji J. Most Cleaved Anti-Müllerian Hormone Binds Its Receptor in Human Follicular Fluid But Little Is Competent in Serum. J Clin Endocrinol Metab (2016) 101:4618–27. doi: 10.1210/jc.2016-1742 27623067

[78] The UniProt Consortium. UniProt: The Universal Protein Knowledgebase in 2021. Nucleic Acids Res (2021) 49:D480–9. doi: 10.1093/nar/gkaa1100 PMC7778908 33237286

[79] Jumper J Evans R Pritzel A Green T Figurnov M Ronneberger O. Highly Accurate Protein Structure Prediction With AlphaFold. Nature (2021) 596:583–9. doi: 10.1038/s41586-021-03819-2 PMC8371605 34265844

[80] Mi L-Z Brown CT Gao Y Tian Y Le VQ Walz T. Structure of Bone Morphogenetic Protein 9 Procomplex. Proc Natl Acad Sci U.S.A. (2015) 112:3710–5. doi: 10.1073/pnas.1501303112 PMC4378411 25751889

[81] Wang X Fischer G Hyvönen M. Structure and Activation of Pro-Activin a. Nat Commun (2016) 7:12052. doi: 10.1038/ncomms12052 27373274 PMC4932183

[82] Cotton TR Fischer G Wang X McCoy JC Czepnik M Thompson TB. Structure of the Human Myostatin Precursor and Determinants of Growth Factor Latency. EMBO J (2018) 37:367–83. doi: 10.15252/embj.201797883 PMC5793801 29330193

[83] Picard J-Y Cate RL Racine C Josso N. The Persistent Müllerian Duct Syndrome: An Update Based Upon a Personal Experience of 157 Cases. Sex Dev (2017) 11:109–25. doi: 10.1159/000475516 28528332

[84] Goebel EJ Hart KN McCoy JC Thompson TB. Structural Biology of the Tgfβ Family. Exp Biol Med (Maywood) (2019) 244:1530–46. doi: 10.1177/1535370219880894 PMC6920667 31594405

[85] Hart KN Stocker WA Nagykery NG Walton KL Harrison CA Donahoe PK. Structure of AMH Bound to AMHR2 Provides Insight Into a Unique Signaling Pair in the TGF-β Family. PNAS (2021) 118:1–10. doi: 10.1073/pnas.2104809118 PMC8256043 34155118

[86] Allendorph GP Vale WW Choe S. Structure of the Ternary Signaling Complex of a TGF-Beta Superfamily Member. Proc Natl Acad Sci U.S.A. (2006) 103:7643–8. doi: 10.1073/pnas.0602558103 PMC1456805 16672363

[87] Groppe J Hinck CS Samavarchi-Tehrani P Zubieta C Schuermann JP Taylor AB. Cooperative Assembly of TGF-Beta Superfamily Signaling Complexes Is Mediated by Two Disparate Mechanisms and Distinct Modes of Receptor Binding. Mol Cell (2008) 29:157–68. doi: 10.1016/j.molcel.2007.11.039 18243111

[88] Weber D Kotzsch A Nickel J Harth S Seher A Mueller U. A Silent H-Bond can be Mutationally Activated for High-Affinity Interaction of BMP-2 and Activin Type IIB Receptor. BMC Struct Biol (2007) 7:6. doi: 10.1186/1472-6807-7-6 17295905 PMC1802081

[89] Radaev S Zou Z Huang T Lafer EM Hinck AP Sun PD. Ternary Complex of Transforming Growth Factor-Beta1 Reveals Isoform-Specific Ligand Recognition and Receptor Recruitment in the Superfamily. J Biol Chem (2010) 285:14806–14. doi: 10.1074/jbc.M109.079921 PMC2863181 20207738

[90] Townson SA Martinez-Hackert E Greppi C Lowden P Sako D Liu J. Specificity and Structure of a High Affinity Activin Receptor-Like Kinase 1 (ALK1) Signaling Complex. J Biol Chem (2012) 287:27313–25. doi: 10.1074/jbc.M112.377960 PMC3431715 22718755

[91] Goebel EJ Corpina RA Hinck CS Czepnik M Castonguay R Grenha R. Structural Characterization of an Activin Class Ternary Receptor Complex Reveals a Third Paradigm for Receptor Specificity. Proc Natl Acad Sci U.S.A. (2019) 116:15505–13. doi: 10.1073/pnas.1906253116 PMC6681762 31315975

[92] Klammert U Mueller TD Hellmann TV Wuerzler KK Kotzsch A Schliermann A. GDF-5 can Act as a Context-Dependent BMP-2 Antagonist. BMC Biol (2015) 13:77. doi: 10.1186/s12915-015-0183-8 26385096 PMC4575486

[93] Keller S Nickel J Zhang J-L Sebald W Mueller TD. Molecular Recognition of BMP-2 and BMP Receptor IA. Nat Struct Mol Biol (2004) 11:481–8. doi: 10.1038/nsmb756 15064755

[94] Kotzsch A Nickel J Seher A Sebald W Müller TD. Crystal Structure Analysis Reveals a Spring-Loaded Latch as Molecular Mechanism for GDF-5-Type I Receptor Specificity. EMBO J (2009) 28:937–47. doi: 10.1038/emboj.2009.37 PMC2670865 19229295

